# Impact of problem-based learning on the development of clinical reasoning skills in esophageal cancer teaching

**DOI:** 10.1097/MD.0000000000047451

**Published:** 2026-02-06

**Authors:** Luo Zhao, Jia He, Zhijun Han, Yisheng Zhang, Li Li

**Affiliations:** a Department of Thoracic Surgery, Peking Union Medical College Hospital, CAMS & PUMC, Dongcheng District, Beijing City, China.

**Keywords:** clinical reasoning, esophageal cancer, medical education, problem-based learning, teaching effectiveness

## Abstract

The complexity of diagnostic and therapeutic decision-making in esophageal cancer underscores the need for effective teaching strategies to enhance clinical reasoning skills. This study evaluated the association of a problem-based learning (PBL) approach with clinical reasoning ability, theoretical knowledge acquisition, and learning perceptions among medical interns. In this retrospective study, 132 medical interns completing thoracic surgery rotations were included. A control group (n = 64) taught via conventional lectures was compared to an observation group (n = 68) instructed using structured PBL modules. Baseline comparability was confirmed. Outcomes included standardized assessments of clinical reasoning performance, diagnostic accuracy, differential diagnosis formulation, and case presentation quality. Theoretical knowledge was tested via written examinations, and self-evaluation questionnaires assessed perceived learning gains. Data were analyzed using Welch Student t test, Mann–Whitney *U* test, and Chi-square tests, with *P* < .05 considered significant. Compared to the control group, the PBL group demonstrated a higher overall clinical reasoning score (87.4 ± 6.3 vs 79.1 ± 7.2; *t* = 7.059, *P* < .001) and greater diagnostic accuracy (92.6% vs 68.8%; *χ*^2^ = 12.264, *P* = .001). The PBL approach was also associated with superior theoretical knowledge scores (85.7 ± 5.9 vs 81.0 ± 6.6; *t* = 4.319, *P* < .001) and higher sub-domain scores in pathophysiology, diagnostic interpretation, and treatment principles (all *P* < .01). Student self-assessments indicated higher satisfaction, stronger self-reported critical thinking, and greater perceived integrative ability in the PBL cohort (all *P* < .001). The structured PBL model was significantly associated with improved clinical reasoning, theoretical knowledge, and self-perceived competence in esophageal cancer education. These findings suggest the potential value of integrating PBL into competency-based medical training for complex oncological diseases.

## 1. Introduction

Esophageal cancer remains one of the most challenging malignancies worldwide, characterized by aggressive biological behavior, late-stage diagnosis, and poor prognosis despite advances in multimodal therapy. The complexity of its diagnostic and therapeutic processes (encompassing endoscopic evaluation, radiologic staging, surgical intervention, chemoradiotherapy, and postoperative management) demands that medical trainees develop advanced clinical reasoning and integrative decision-making skills early in their education. Effective teaching models are thus critical to cultivate not only theoretical understanding but also the cognitive frameworks required for evidence-based clinical practice in esophageal oncology.^[1,2]^ Traditional lecture-based teaching remains the predominant mode of medical instruction in many institutions. Although effective for conveying factual and conceptual knowledge, this method often promotes passive learning and limits opportunities for hypothesis-driven inquiry, analytic thinking, and problem-solving. In contrast, problem-based learning (PBL), first introduced by Barrows and Tamblyn, emphasizes active engagement, small-group collaboration, and self-directed exploration of clinical problems that mirror real-world scenarios.^[3,4]^ Through exposure to authentic clinical cases, students formulate learning objectives, generate differential diagnoses, and apply multidisciplinary knowledge to reach reasoned conclusions. Such an approach is particularly relevant to esophageal cancer education, where diagnosis and management frequently require the synthesis of complex clinical, endoscopic, and pathological data.

Emerging evidence suggests that PBL can effectively enhance higher-order cognitive competencies, including diagnostic reasoning, critical appraisal, and integrative learning. Recent meta-analyses in medical education have demonstrated that PBL improves students’ problem-solving ability, long-term knowledge retention, and satisfaction compared with conventional didactic methods. In clinical disciplines, PBL has been associated with better application of guideline-based decision-making and improved adaptability to complex patient scenarios.^[5,6]^ Studies within oncology education have similarly shown that case-based or problem-centered learning facilitates deeper comprehension of pathophysiological mechanisms and promotes reasoning applicable to tumor staging, treatment planning, and prognostic evaluation. However, despite the growing adoption of PBL in various medical fields, its structured application in esophageal cancer teaching remains limited, and few studies have systematically quantified its effect on the development of clinical reasoning and related cognitive domains among medical interns. Clinical reasoning represents the cornerstone of medical expertise and involves an iterative process of data gathering, hypothesis generation, and diagnostic synthesis. It integrates biomedical knowledge with contextual patient information to formulate accurate diagnoses and management strategies. Deficiencies in reasoning contribute to diagnostic error and suboptimal patient care.^[7–9]^ Therefore, optimizing instructional strategies that enhance reasoning processes is of paramount importance, especially in domains like thoracic oncology where diagnostic uncertainty and therapeutic complexity are prevalent. PBL, by stimulating contextualized reasoning and reflection, may bridge the gap between theoretical instruction and clinical application.

Given this pedagogical context, the present study was designed to evaluate the educational impact of PBL in esophageal cancer teaching among medical interns. Specifically, it assessed whether a structured PBL model could improve clinical reasoning performance, enhance theoretical knowledge acquisition, and promote self-perceived learning outcomes compared with traditional lecture-based instruction. By examining both objective and subjective indicators, this study aimed to provide evidence supporting the integration of PBL as an effective, competency-oriented instructional framework in medical education for complex oncologic diseases such as esophageal cancer.

## 2. Methods

### 2.1. Study design

This study was approved by the Ethics Committee of Peking Union Medical College Hospital. This retrospective study enrolled medical interns who completed clinical rotations in the Department of Thoracic Surgery at our institution between September 2023 and June 2025. Interns trained between September 2023 and June 2024 received conventional teacher-centered instruction and were designated as the control group, whereas interns rotating between September 2024 and June 2025 were instructed using a PBL approach and constituted the observation group. Eligible participants were medical students undertaking full-time clinical internship in esophageal cancer teaching modules, who completed the entire teaching cycle and participated in all required theoretical and practical assessments. Exclusion criteria included incomplete internship duration, prior exposure to PBL-based thoracic oncology teaching, refusal to participate, or missing essential evaluation data. The study procedures followed the ethical principles outlined in the Declaration of Helsinki, and Institutional Ethics Committee approval was obtained prior to study initiation. Written informed consent was obtained from all interns before data collection. A concurrent randomized controlled trial was not feasible due to the pragmatic constraints of the standardized, cohort-based internship curriculum at our institution, which allocates all students in a given academic year to the same teaching model.

### 2.2. Intervention protocols

Control group (Conventional Teaching Model): interns assigned to the control group received conventional teacher-centered teaching throughout the esophageal cancer clinical teaching curriculum. Instruction was delivered primarily in a lecture-based format, supported by standardized multimedia slides, structured didactic explanation of disease pathophysiology, diagnostic pathways, treatment strategies, surgical indications, and postoperative management principles. Clinical case exposure occurred in ward rounds led by senior attending physicians, primarily in a unidirectional explanation mode. Students passively received clinical knowledge supplemented by instructor demonstrations; problem formulation, hypothesis generation, group discussion, and independent evidence retrieval were not systematically required. Routine after-class theoretical review and written examinations were utilized for performance feedback.

Observation group (Problem-Based Learning Teaching Model): interns in the observation group underwent a structured PBL intervention framework during the same esophageal cancer teaching module. Students were divided into small fixed learning groups (6–8 interns per group). Each teaching unit began with real clinical case scenarios involving esophageal cancer patients, including initial clinical presentation, diagnostic imaging, endoscopy findings, and therapeutic decision points. Students were required to identify learning objectives, actively retrieve supporting evidence from guideline-based literature, develop diagnostic reasoning chains, and propose differential diagnosis and management strategies through iterative group discussion. The instructor served as a facilitator to guide cognitive reasoning and ensure accuracy of clinical conclusions without providing prestructured answers. Each module concluded with group oral reporting, peer feedback, reflective discussion, and formative assessment focusing on clinical reasoning development rather than memory-based recall.

### 2.3. Data collection

Two independent investigators extracted and cross-checked the data using a standardized case report form to ensure completeness and consistency. The collected information included demographic characteristics (age and sex), baseline academic records (undergraduate major, pre-rotation theoretical examination scores), and details of internship participation (rotation period, attendance rate, and completion of assessments). Educational performance indicators were systematically obtained from the evaluation records of each teaching module. These included clinical reasoning assessments (comprising overall reasoning score, diagnostic accuracy, differential diagnosis formulation, therapeutic decision-making consistency, and case presentation quality); theoretical knowledge examinations (including total and sub-domain scores for pathophysiology, diagnostic interpretation, and treatment principles); and self-evaluation questionnaires, covering perceived self-efficacy, integrative analytical ability, satisfaction with instructional methods, and improvement in critical thinking. All data were verified against original evaluation forms and examination records. Discrepancies between reviewers were resolved through consensus. The accuracy of data entry was validated by random double-checking of 20% of the cases. For the objective assessments (clinical reasoning and theoretical exams), grading was performed by 2 independent assessors who were blinded to the student group allocation (PBL or control) during the scoring process.

### 2.4. Statistical analysis

All statistical analyses were performed using IBM Statistical Package for the Social Sciences Statistics, version 26.0 (IBM Corp., Armonk). Continuous variables were tested for normality using the Kolmogorov–Smirnov test. Normally distributed data were expressed as mean ± standard deviation and compared between groups using the independent-samples Student t test (*t* test) (Welch *t* test). Non-normally distributed variables were presented as median and interquartile range (IQR) and analyzed using the Mann–Whitney *U* test. Categorical variables were described as frequency and percentage (%) and compared using the Chi-square test (*χ*^2^) or Fisher exact test when appropriate. All statistical tests were two-tailed, and a *P*-value <.05 was considered statistically significant.

## 3. Results

### 3.1. Baseline characteristics

A total of 132 medical interns were included (control, n = 64; PBL, n = 68). Mean age was 21.8 ± 1.5 years in the control group and 22.1 ± 1.7 years in the PBL group; the difference was not statistically significant (Welch *t* = 1.072, two-tailed *P* = .285). Sex distribution was comparable (male/female: 30/34 vs 28/40; *χ*^2^ = 0.435, *P* = .510). All interns held a Bachelor’s degree. Baseline theoretical examination scores did not differ significantly (76.8 ± 6.5 vs 78.2 ± 7.2; Welch *t* = 1.170, *P* = .244). These data indicate no significant between-group differences at study entry (Table 1).

**Table 1:** Demographic and clinical characteristic.

Variable	Control (n = 64)	PBL (n = 68)	Total (n = 132)	Test statistic	*P* value
Age (yr) (mean ± SD)	21.8 ± 1.5	22.1 ± 1.7	22.0 ± 1.6	*t* = 1.072	.285
Sex, male/female, n (%)	30 (46.9)/34 (53.1)	28 (41.2)/40 (58.8)	58 (43.9)/74 (56.1)	*χ*^2^ = 0.435	.510
Academic background, Bachelor’s, n (%)	64 (100.0)	68 (100.0)	132 (100.0)	–	–
Baseline theoretical exam (0–100), mean ± SD	76.8 ± 6.5	78.2 ± 7.2	77.5 ± 6.9	*t* = 1.170	.244

PBL = problem-based learning, SD = standard deviation.

### 3.2. Clinical reasoning assessment outcomes

After completion of the esophageal cancer teaching module, the PBL group demonstrated significantly superior performance across all clinical reasoning domains when compared with the control group. The overall clinical reasoning performance score was higher in the PBL group (mean ± standard deviation, 87.4 ± 6.3) relative to the control group (79.1 ± 7.2), Welch *t* = 7.059, *P* < .001. Diagnostic reasoning accuracy was also higher in the PBL cohort (63/68, 92.6%) compared with the control cohort (44/64, 68.8%), *χ*^2^ = 12.2641, *P* = .001. The PBL group demonstrated a greater number of correctly formulated differential diagnosis items (median 8, IQR 7–9) than the control group (median 6, IQR 5–7); Mann–Whitney *U* = 968, *z* = 5.50, *P* < .001, with a Hodges–Lehmann median difference of 2 items. Therapeutic decision-making consistency with guideline-based standards was higher in the PBL cohort (58/68, 85.3%) relative to the control cohort (39/64, 60.9%), *χ*^2^ = 10.038, *P* = .002. In addition, structured case presentation quality scores were higher in the PBL group (88.6 ± 5.4) compared with the control group (80.2 ± 6.8), Welch *t* = 7.883, *P* < .001 (Table 2).

**Table 2:** Clinical reasoning assessment outcomes.

Outcome	Control (n = 64)	PBL (n = 68)	Effect metric	Test statistic	*P* value
Overall clinical reasoning score (0–100), mean ± SD	79.1 ± 7.2	87.4 ± 6.3	Mean difference = 8.3	Welch *t* = 7.059	<.001
Diagnostic reasoning accuracy, n/N (%)	44/64 (68.8)	63/68 (92.6)	Risk difference = 23.8%	*χ*^2^ = 12.2641	.001
Correct items in differential diagnosis list, median (IQR)	6 (5–7)	8 (7–9)	HL median difference = 2	Mann–Whitney *U* = 968; *z* = 5.50	<.001
Therapeutic decision-making consistent with guidelines, n/N (%)	39/64 (60.9)	58/68 (85.3)	Risk difference = 24.4%	*χ*^2^ = 10.038	.002
Structured case presentation quality (0–100), mean ± SD	80.2 ± 6.8	88.6 ± 5.4	Mean difference = 8.4	Welch *t* = 7.883	<.001

PBL = problem-based learning, SD = standard deviation.

### 3.3. Theoretical knowledge examination

Following completion of the teaching rotation, the PBL group demonstrated significantly superior theoretical knowledge outcomes compared with the control group. The mean end-of-rotation theoretical examination score was higher in the PBL cohort (85.7 ± 5.9) than in the control cohort (81.0 ± 6.6), Welch *t* = 4.319, *P* < .001. Sub-domain analysis showed that the PBL group achieved higher performance in pathophysiological understanding of esophageal cancer (27.6 ± 2.8 vs 25.9 ± 3.4; Welch *t* = 3.143, *P* = .002), diagnostic interpretation (28.4 ± 3.1 vs 26.3 ± 3.5; Welch *t* = 3.654, *P* < .001), and treatment principle mastery (29.7 ± 2.6 vs 28.0 ± 3.1; Welch *t* = 3.421, *P* = .001). The proportion of students achieving high theoretical performance (defined as ≥85 points) was higher in the PBL group (46/68, 67.6%) relative to the control group (28/64, 43.8%), *χ*^2^ = 7.643, *P* = .006. These results indicate that PBL not only improved overall theoretical knowledge level but also enhanced key cognitive components relevant to esophageal cancer clinical management (Table 3).

**Table 3:** Theoretical knowledge examination outcomes.

Outcome Variable	Control (n = 64)	PBL (n = 68)	Effect metric	Test statistic	*P* value
Total theoretical exam score (0–100), mean ± SD	81.0 ± 6.6	85.7 ± 5.9	Mean difference = 4.7	Welch *t* = 4.319	<.001
Pathophysiology sub-score (0–30), mean ± SD	25.9 ± 3.4	27.6 ± 2.8	Mean difference = 1.7	Welch *t* = 3.143	.002
Diagnostic interpretation sub-score (0–30), mean ± SD	26.3 ± 3.5	28.4 ± 3.1	Mean difference = 2.1	Welch *t* = 3.654	<.001
Treatment principle sub-score (0–30), mean ± SD	28.0 ± 3.1	29.7 ± 2.6	Mean difference = 1.7	Welch *t* = 3.421	.001
High achievement ≥ 85 points, n/N (%)	28/64 (43.8%)	46/68 (67.6%)	Risk difference = 23.8%	*χ*^2^ = 7.643	.006

PBL = problem-based learning, SD = standard deviation.

### 3.4. Student self-evaluation of learning outcomes

Self-evaluation questionnaires demonstrated that interns in the PBL group reported significantly higher perceived learning gains across multiple subjective learning dimensions compared with the control group. The PBL cohort reported higher perceived self-efficacy in analyzing complex esophageal cancer cases (8.6 ± 1.0 vs 7.2 ± 1.3; Welch *t* = 6.959, *P* < .001), identifying key diagnostic cues (8.4 ± 1.1 vs 7.1 ± 1.4; Welch *t* = 5.951, *P* < .001), and interpreting imaging and pathology information (8.5 ± 1.0 vs 7.3 ± 1.3; Welch *t* = 5.965, *P* < .001). In addition, the PBL group demonstrated higher perceived capacity in integrating multidimensional clinical information (8.7 ± 1.0 vs 7.4 ± 1.2; Welch *t* = 6.777, *P* < .001). Overall instructional satisfaction was higher in the PBL cohort (8.9 ± 0.9) compared with the control group (7.6 ± 1.2), Welch *t* = 7.068, *P* < .001, and perceived improvement in independent critical thinking ability was also greater (8.8 ± 1.0 vs 7.5 ± 1.3), Welch *t* = 6.462, *P* < .001. These findings support that PBL provided broader subjective educational benefit beyond objective knowledge acquisition (Table 4).

**Table 4:** Student self-evaluation of learning outcomes.

Outcome (0–10 Likert scale)	Control (n = 64)	PBL (n = 68)	Effect metric	Test statistic	*P* value
Self-efficacy in analyzing complex cases	7.2 ± 1.3	8.6 ± 1.0	Mean difference = 1.4	Welch *t* = 6.959	<.001
Identification of key diagnostic cues	7.1 ± 1.4	8.4 ± 1.1	Mean difference = 1.3	Welch *t* = 5.951	<.001
Imaging and pathology interpretation	7.3 ± 1.3	8.5 ± 1.0	Mean difference = 1.2	Welch *t* = 5.965	<.001
Multidimensional clinical information integration	7.4 ± 1.2	8.7 ± 1.0	Mean difference = 1.3	Welch *t* = 6.777	<.001
Overall instructional satisfaction	7.6 ± 1.2	8.9 ± 0.9	Mean difference = 1.3	Welch *t* = 7.068	<.001
Perceived improvement in independent critical thinking	7.5 ± 1.3	8.8 ± 1.0	Mean difference = 1.3	Welch *t* = 6.462	<.001

PBL = problem-based learning.

## 4. Discussion

This study evaluated the effect of a structured PBL model on the development of clinical reasoning skills within an esophageal cancer teaching module for medical interns. Across objective performance measures and subjective evaluations, interns exposed to PBL demonstrated higher overall clinical reasoning scores, greater diagnostic accuracy, more comprehensive differential diagnosis lists, and greater concordance of therapeutic decisions with guideline-based standards. PBL was also associated with higher theoretical examination scores and superior sub-domain knowledge in pathophysiology, diagnostic interpretation, and treatment principles. Student self-evaluation indicated higher perceived ability to analyze complex cases, identify key diagnostic cues, interpret imaging and pathology findings, integrate multisource clinical information, and apply independent critical thinking. The pattern of effects, simultaneous gains in reasoning, knowledge, and self-efficacy, suggests that PBL promotes the integration of declarative knowledge with procedural and metacognitive competencies that are central to clinical decision-making.

From an instructional design perspective, the observed differences are consistent with mechanisms proposed for PBL. First, authentic cases require learners to externalize problem representations, generate and test diagnostic hypotheses, and iteratively reconcile discrepancies using targeted information retrieval. This sequence mirrors clinical reasoning processes such as problem formulation, illness script activation, and data interpretation, plausibly which may plausibly account for the higher diagnostic accuracy and more extensive differential lists in the PBL cohort. Second, small-group discourse and instructor facilitation may have supported deliberate practice in argumentation and justification, which aligns with the observed gains in guideline-concordant therapeutic choices and case presentation quality. Third, the mapping between learning objectives and sub-domain outcomes indicates that structured case prompts and literature-based tasks can expand domain-specific knowledge while maintaining a focus on reasoning performance.^[10,11]^

The theoretical examination results merit emphasis. Although PBL is designed to cultivate reasoning and self-directed learning rather than rote recall, the PBL cohort also achieved higher scores on a standardized written assessment and outperformed the control group across knowledge sub-domains. This suggests that, in this setting, reasoning-oriented inquiry did not trade off against content mastery; instead, exposure to problems that require explanation, mechanism, and evidence appraisal may have promoted deeper encoding and retrieval. The higher proportion of students meeting the high-achievement threshold (≥85 points) further indicates that PBL’s benefit was not limited to a small subset of learners. Self-evaluation data showed consistent, domain-relevant improvements that paralleled objective outcomes. Increases in perceived capability for complex case analysis, cue identification, and multimodal data integration correspond to the PBL tasks that repeatedly required learners to synthesize clinical history, endoscopic findings, imaging, and pathology within the same case. While subjective measures are vulnerable to bias, the convergence of subjective and objective indicators strengthens the inference that the PBL model was linked to enhancements in both competence and confidence in clinically salient skills.^[12]^

Recent evidence in medical education supports the effectiveness of PBL for cultivating higher-order competencies, including critical thinking and clinical skills. A 2025 scoping review of PBL across health professions reported positive associations with critical thinking, clinical skill development, and learner engagement, noting that case-driven small-group formats can improve analytic performance and transfer to clinical tasks.^[13]^ Similarly, a 2025 meta-analytic review concluded that PBL outperforms conventional teaching in enhancing critical thinking among medical students, reinforcing the link between problem-driven learning and reasoning outcomes.^[14]^ Findings in oncology-focused education also align with the present results. In radiation oncology, a 2024 study that implemented a PBL model on a digital platform reported improved resident engagement and knowledge outcomes, suggesting that problem-centered cases in oncologic contexts facilitate integration of complex diagnostic and therapeutic content.^[15]^ Outside radiation oncology, case- or problem-based approaches in cancer education have shown advantages over traditional lectures for knowledge and application, as illustrated by a 2024 comparison of case-based learning versus conventional teaching in advanced cancer modules, which favored the active learning approach on multiple assessment endpoints.^[16]^

Assessment research underscores that performance-based measures (e.g., objective structured clinical examination [OSCEs], structured oral or case presentations) capture facets of reasoning that written tests may miss. A 2023 scoping review on OSCE comparability highlighted the value of multimodal assessments for evaluating clinical competence, supporting the inclusion of case presentation quality and guideline-concordance as meaningful outcomes in studies of educational interventions.^[17]^ Additionally, recent work proposing innovative methods to profile clinical reasoning components in senior medical students echoes our focus on diagnostic hypothesis generation and data interpretation as discrete skill targets.^[18]^ Finally, synthesis papers mapping global trends in PBL emphasize competency-based curricula and integration of evidence-based reasoning early in training (directions consistent with our module’s design and observed benefits).^[19]^ Taken together, these contemporary sources converge with our results: PBL, when implemented with authentic cases, facilitator guidance, and structured assessment, yields measurable gains in clinical reasoning and related knowledge in oncologic teaching settings.

Esophageal cancer care requires integration of symptom appraisal, risk stratification, multimodal imaging, endoscopic evaluation, pathology interpretation, and stage-adapted treatment planning. The observed PBL-associated gains in diagnostic accuracy, differential diagnosis breadth, and guideline-concordant decision-making indicate that problem-centered curricula can better prepare interns to navigate these integrative demands. Embedding PBL cases that mirror real decision points may shorten the transition from classroom to ward-based reasoning, improve pre-round preparation and case synthesis, and support safer, more consistent management aligned with contemporary guidelines. Programs teaching other thoracic or gastrointestinal malignancies may consider analogous PBL structures to strengthen reasoning and knowledge integration. This study has several strengths. First, it evaluated a comprehensive set of outcomes spanning objective performance (reasoning score, diagnostic accuracy, differential diagnosis count, guideline concordance, and case presentation quality), knowledge (total and sub-domain written scores), and learner-reported indicators (self-efficacy, satisfaction, perceived critical thinking). The triangulation of measures reduces single-method bias and provides a multidimensional view of educational impact. Second, the PBL intervention was defined a priori with fixed group sizes and standardized case prompts, improving instructional fidelity. Third, analyses used appropriate tests for data type and distribution, with effect metrics reported alongside test statistics to facilitate interpretation.

Several limitations warrant caution. The retrospective, nonrandomized design introduces potential selection and cohort effects; although baseline comparability was acceptable, unmeasured confounders cannot be excluded. The study was conducted at a single institution, which may limit generalizability to programs with different learner profiles or resource availability. Theoretical examinations may not fully capture applied reasoning; while case presentation and guideline-concordance partially address this, future studies should incorporate performance-based assessments such as OSCEs mapped to the same competencies. Self-evaluation outcomes are subject to response and acquiescence biases; triangulation with external ratings or workplace-based assessments (e.g., Mini-Clinical Evaluation Exercise) would strengthen inferences. Finally, sustainability and scalability were not assessed; implementation research is needed to determine resource requirements, faculty development needs, and long-term retention of PBL-acquired skills. Second, despite demonstrating comparability in recorded baseline characteristics (age, sex, pre-rotation scores), the possibility of residual confounding from unmeasured or unrecorded factors (e.g., inherent motivation, prior informal exposure to case-based learning, differential engagement in self-study) cannot be excluded. These factors might independently influence both learning outcomes and responsiveness to the PBL method. Third, although we implemented measures to ensure consistency in faculty, guidelines, and assessment blueprints, the non-randomized, retrospective cohort design based on sequential academic years remains susceptible to unmeasured temporal confounding. Factors such as subtle yearly variations in the overall intern cohort preparedness, external educational exposures, or unrecorded minor adjustments to clinical case availability could have influenced the outcomes.

## 5. Conclusions

PBL significantly enhanced medical interns’ clinical reasoning, theoretical knowledge, and self-perceived learning outcomes in esophageal cancer education. Compared with conventional instruction, PBL improved diagnostic accuracy, differential diagnosis formulation, guideline-concordant decision-making, and theoretical understanding across multiple cognitive domains. These findings support incorporating structured PBL modules into esophageal cancer teaching to strengthen integrative reasoning and promote competency-based clinical training.

## Author contributions

**Conceptualization:** Luo Zhao, Jia He, Zhijun Han, Yisheng Zhang, Li Li.

**Data curation:** Luo Zhao, Jia He, Zhijun Han, Yisheng Zhang, Li Li.

**Formal analysis:** Luo Zhao, Jia He, Zhijun Han, Yisheng Zhang, Li Li.

**Funding acquisition:** Li Li.

**Investigation:** Li Li.

**Writing – original draft:** Luo Zhao, Zhijun Han, Li Li.

**Writing – review & editing:** Luo Zhao, Zhijun Han, Li Li.
